# Global Trends in Incidence and Burden of Urolithiasis from 1990 to 2019: An Analysis of Global Burden of Disease Study Data

**DOI:** 10.1016/j.euros.2021.10.008

**Published:** 2022-01-03

**Authors:** Jacob Lang, Aparna Narendrula, Ahmed El-Zawahry, Puneet Sindhwani, Obi Ekwenna

**Affiliations:** a University of Toledo College of Medicine and Life Sciences, Toledo, OH, USA; b Case Western Reserve University School of Medicine, Cleveland, OH, USA; c Department of Urology and Transplantation, University of Toledo College of Medicine and Life Sciences, Toledo, OH, USA

**Keywords:** Urolithiasis, Epidemiology, Global health, Disease burden, Quality of life

## Abstract

**Background:**

Urolithiasis is among the most common urologic diagnoses globally, with substantial burden and cost on healthcare systems worldwide. Increasing evidence links urolithiasis with an array of risk factors, including diet and lifestyle trends, noncommunicable diseases such as diabetes and obesity, and global warming.

**Objective:**

To examine geographic, temporal, and sociodemographic patterns to better understand global disease burden of urolithiasis.

**Design, setting, and participants:**

We extracted data on age-standardized incidence rate (ASIR), deaths, and disability-adjusted life years (DALYs) attributed to urolithiasis for 21 regions, including 204 countries, for 1990–2019 from the Global Burden of Disease (GBD) study.

**Outcome measurements and statistical analysis:**

Data were analyzed at the global, regional, and country levels, as well as stratified by the Socio-Demographic Index. The average annual percentage change (AAPC) was calculated to measure temporal trends across groups.

**Results and limitations:**

Globally, total cases, DALYs, and deaths attributed to urolithiasis increased over the study period, while the age-standardized rates of these measures decreased. The age-standardized incidence of urolithiasis decreased from 1696.2 (95% confidence interval [CI] 1358.1–2078.1) cases per 100 000 population in 1990 to 1394.0 (95% CI, 1126.4–1688.2) cases per 100 000 population in 2019, with an AAPC of −0.7 (95% CI [−0.8, −0.6]). Of the GBD regions, Eastern Europe demonstrated a consistently higher ASIR of urolithiasis than all other regions, while the Caribbean had the highest AAPC. This study is limited by the available national and regional data, as described in the original GBD study.

**Conclusions:**

Worldwide, total cases, DALYs, and deaths attributed to urolithiasis have increased since 1990, while age-standardized rates have decreased, with demonstrated regional and sociodemographic variation. Multifaceted strategies to address urolithiasis prevention and treatment are necessary.

**Patient summary:**

In this study, we looked at trends in the global burden of stone disease using data from 204 countries from 1990 to 2019. We found that the overall burden has increased, but it varies by age, sociodemographic variables, and geographic region. We conclude that we need adaptable policies that suit the specific needs of the country to address this burden.

## Take Home Message

The total burden of urolithiasis in terms of incidence, disability-adjusted life years, and deaths has increased globally since 1990, while age-standardized rates have decreased. Importantly, areas of lower sociodemographic index have seen an increased burden. Understanding variation and factors affecting the burden of urolithiasis is necessary for policymakers.

## 1 Introduction

Urolithiasis is one of the most common urologic diseases worldwide, with an estimated prevalence ranging from 1% to 13% in different regions across the globe [1], [2]. Recent evidence demonstrates that the prevalence of urolithiasis is on the rise globally due to a multitude of factors, including changes in social conditions, dietary habits, climate, and disease comorbidities [1], [3], [4], [5]. With this change comes increases in disease burden, associated costs of diagnosis and treatment borne by healthcare systems, and economic burden due to the deleterious effects of urolithiasis [3], [6], [7].

While the burden of urolithiasis is universally increasing, the epidemiology of urolithiasis varies across regions of the globe [8]. The societal cost of a case of urolithiasis varies by region, but saliently the trends in incidence and prevalence vary in conjunction with imbalances in economic development, obesity rates, diet, climate change, and other health conditions. Thus, measuring and effectively addressing the burden of urolithiasis require a culturally and geographically adapted approach. Few global and national estimates and reviews describing this varying burden exist, meriting a comprehensive global comparison of the incidence, disability burden, and mortality associated with urolithiasis [9], [10].

The Global Burden of Disease (GBD) 2019 study provides a systematic assessment of published and publicly available evidence of incidence, prevalence, and mortality for 369 diseases and injuries between 1990 and 2019 for 204 countries and territories and 21 regions [11]. However, no analyses highlighting and analyzing the trends and incidence of urolithiasis specifically using GBD study data have been published previously. Using estimates of the disease burden of urolithiasis provided by the GBD study, this study aims to describe and analyze global, regional, and national epidemiologic trends and disease burden of urolithiasis from 1990 to 2019 in order to better understand and address the burden of urolithiasis going forward.

## 2 Patients and methods

Data on the age-standardized incidence rate (ASIR) per 100 000 population, age-standardized rate (ASR) of disability-adjusted life years (DALYs) estimated based on the addition of the years lived with disability and the years of life lost, and age-standardized death rate (ASDR), as well as the total for these measures, attributed to urolithiasis were available from the publicly available Global Health Data Exchange (GHDx) query tool [12]. These measures are considered the objective index in understanding trends related to disease occurrence and burden. The GBD study provides estimates of incidence, prevalence, DALYs, and other health indicators for 369 diseases and injuries. Detailed methods of the GBD study have been described previously [11], [13], [14]. Data from the GBD study on urolithiasis were collected from national and international vital registries of hospital claims and outpatients data, verbal autopsy data, and a systematic literature review. Estimates by age, sex, year, and country were calculated using a Bayesian meta-regression modeling tool, DisMod-MR 2.1, for consistency [15].

We extracted case data for all ages and annual ASRs of these measures of urolithiasis from 1990 to 2019 for 21 regions, including 204 countries and territories. Data were analyzed at the global and regional levels, as well as stratified by the Socio-Demographic Index (SDI), which is based on national income per capita, average years of education of adults, and total fertility rate, to assess geographic and socioeconomic trends.

The average annual percentage change (AAPC) was calculated at the global, regional, and national levels as a summary statistic for the trends in ASRs of incidence, DALYs, and deaths. AAPC is a single number that describes disease occurrence in a population by using the weighted averages of annual percent changes [16]. To calculate the AAPC, Joinpoint Trend Analysis software was used to estimate an underlying model with the best fit for each region’s ASRs for urolithiasis. The AAPC of each interval is calculated as the weighted average of the slope of the underlying Joinpoint linear regression lines. This weighted average of slopes is then converted to an annual percentage change. Joinpoint developed a model for each country that combined the best fit of varying numbers of linear regressions *y* = *b0* + *b1x* + *c* such that *y* = *ln(ASR)* and *x* = calendar year. The AAPC is then reported as 100 × [exp(*b1*) – 1] with its respective 95% confidence interval (CI) [17].

Generalized additive modeling was then used to demonstrate the relationship of country AAPCs with SDI in 2019, which is used as a surrogate for current country socioeconomic profile, as well as with ASRs in 1990 in order to compare the influence of baseline ASRs on change over the study period. Generalized additive modeling is used widely for time series data in health and allows for incorporation of nonlinear relationships into the linear model framework [18], [19], [20]. Pearson’s correlation coefficient and *p* value were also calculated to determine directionality and significance of the relationship. Significance was determined at the *p* < 0.05 level.

Statistical analyses were performed using R (R Foundation for Statistical Computing, Vienna, Austria) and Joinpoint Trend Analysis software (National Cancer Institute, Bethesda, Maryland), while data visualization was performed in R (R Foundation for Statistical Computing) and Tableau Software (Tableau Software, Seattle, Washington). The GBD study follows the Guidelines for Accurate and Transparent Health Estimates Reporting (GATHER) for population health research. This study uses publicly available data from the GHDx query tool without personal identifiers and was considered exempt from University of Toledo Institutional Review Board review.

## 3 Results

### 3.1 Current burden of urolithiasis

In 2019, 115 552 140 incident cases (95% CI [93 045 130.4–140 180 402.4]) of urolithiasis with 604 308.9 attributed DALYs (95% CI [477 353.5–745 193.9]) and 13 278.9 deaths (95% CI [10 616.0–16 267.4]) occurred globally (Supplementary Tables 1 and 2). Over one-fifth of all incident cases in 2019 occurred in India (25 291 358.9; 95% CI [19 882 953.8–31 444 662.0]), followed by China (17 684 919.0; 95% CI [14 099 066.0–21 623 473.7]) and the Russian Federation (9 060 658.47; 95% CI [7 277 388.1–11 110 813.1]). The distribution of total DALYs attributed to urolithiasis followed a similar pattern to that in India, followed by China and the Russian Federation demonstrating the highest burden, while China demonstrated the highest burden of death, followed by India and the Russian Federation.

When examining ASRs per 100 000 population, the global ASIR of urolithiasis in 2019 was 1394 (95% CI, 1126.4–1688.2; Table 1), with the highest rates occurring in the Russian Federation (4541.9; 95% CI [3648.9–5522.0]), followed by Ukraine (4282.6; 95% CI [3,377.6–5271.8]) and Latvia (4156.7; 95% CI [3404.7–5049.0]), while the lowest rates occurred in Burundi (525.01; 95% CI [408.4–646.9]) followed by South Sudan (533.4; 95% CI [416.2–657.5]). The highest ASR per 100 000 population of DALYs in 2019 occurred in Armenia (33.3; 95% CI [21.7–61.3]) followed by the Russian Federation (24.7; 95% CI [19.7–30.6]), while the lowest ASR of DALYs occurred in Cabo Verde (2.3; 95% CI [1.5–3.2]). ASRs of death attributed to urolithiasis were generally less than one per 100 000 population, with only Armenia surpassing this mark (1.8; 95% CI [0.9–4.0)]).

**Table 1:** Incident cases, ASIRs, and AAPC of urolithiasis in 1990 and 2019 globally as well as among SDI quintile and 21 GBD regions

Group	1990		2019		1990–2019
	Incident cases, number × 10 (95% CI)	ASIR per 100 000 (95% CI)	Incident cases, number × 10^3^ (95% CI)	ASIR per 100 000 (95% CI)	AAPC (95% CI)
Global	77 775.8 (62 239.1–95 126.8)	1696.2 (1358.1–2078.1)	115 552.1 (93 045.1–140 180.4)	1394 (1126.4–1688.2)	−0.7 (−0.8, −0.6)
SDI					
High SDI	14 595.5 (11 509.2–18 009.6)	1556.7 (1228–1924.4)	17 525 (14 314.9–21 186.1)	1288.7 (1053.9–1544.1)	−0.6 (−0.8, −0.5)
High-middle SDI	26 166.5 (20 926.2–32 001.1)	2273.3 (1819.8–2776.7)	29 432.8 (23 329.1–35 943.2)	1576.4 (1268.9–1918.4)	−1.3 (−1.3, −1.2)
Middle SDI	21 117.1 (16 756.1–25 915.1)	1582.7 (1255.2–1938.7)	33 594.2 (26 948.1–41 043.7)	1242.7 (1000.9–1510.6)	–0.8 (−1.1, −0.5)
Low-middle SDI	12 447.7 (9993.7–15 275)	1485.6 (1193.4–1813.8)	24 292.3 (19 365.6–29 869.5)	1460.6 (1159.3–1788.4)	0 (−0.1, 0)
Low SDI	3419.3 (2698.1–4207.1)	954.9 (755.2–1176.7)	7870.9 (6191.4–9729.7)	981.9 (771.3–1212.3)	0.1 (0, 0.3)
Region					
Andean Latin America	459.2 (366.7–571.1)	1609.5 (1290.3–1977.8)	1107.3 (916.9–1323.9)	1772.4 (1472.6– 2110.7)	0.4 (0.3, 0.4)
Australasia	314 (246.6–387.2)	1405.3 (1096.4–1739.1)	477 (373.8–589.8)	1283.4 (1004.7– 1573.7)	−0.3 (−0.4, −0.3)
Caribbean	314.1 (247.9–386.8)	1056.5 (830.2–1310.5)	631.9 (496–788.2)	1239.7 (979.3–1540.4)	0.6 (0.5, 0.6)
Central Asia	1014.6 (805.2–1240.7)	1755.5 (1403.6–2151)	1655.3 (1315.1–2032)	1788 (1435.5–2174.9)	0.1 (0, 0.1)
Central Europe	2320.3 (1830.2–2855.5)	1657.2 (1324.6–2032.8)	1773.6 (1461.2–2143.9)	1178.9 (977.1–1401)	−1.1 (−1.3, −1)
Central Latin America	1117 (887.5–1371.5)	974.9 (774.1–1202.2)	2583.8 (0–3127.3)	1012.4 (810.4–1222.6)	0.1 (0, 0.2)
Central Sub-Saharan Africa	199 (156.3–246.6)	533.2 (417.7–663.5)	531.6 (413.1–657.2)	575.4 (446.6–711.2)	0.3 (0.2, 0.3)
East Asia	16 861.9 (13 224.2–20 887.7)	1592.8 (1245.3–1984.7)	18 531.4 (14 785.9–22 669.2)	901.8 (727.3–1088.8)	−2 (−2.2, −1.8)
Eastern Europe	13 876.9 (11 239.2–16 815.6)	5143.8 (4155.8–6201.3)	12 733.9 (10 201.4–15 601.1)	4433.7 (3542.5–5414.7)	–0.5 (−0.6, −0.4)
Eastern Sub-Saharan Africa	648 (514.6–794.8)	548.6 (432–674.4)	1543.3 (1213.3–1904)	565.7 (444.3–692.3)	0.1 (0.1, 0.2)
High-income Asia Pacific	3123.7 (2382.2–3929.7)	1536.4 (1181.3–1920.9)	3947 (3161.1–4835.9)	1475.2 (1172.9–1795.9)	−0.1 (−0.2, −0.1)
High-income North America	5060.4 (3964.9–6295.3)	1621 (1270.2–2010.7)	4740.2 (4069.4–5561.8)	982.9 (843.8–1137.4)	−1.7 (−1.8, −1.7)
North Africa and Middle East	2913.7 (2281.7–3615.3)	1159.4 (904.4–1445.6)	7448.2 (5834.8–9354.2)	1250.7 (985.9–1553.3)	0.3 (0.2, 0.3)
Oceania	44.1 (34.4–55.4)	978.4 (758.6–1220.2)	109.7 (84.8–138.9)	1033.1 (799.5–1296.2)	0.2 (0.2, 0.3)
South Asia	13 037.4 (10 272.8–16 212.6)	1518 (1206.4–1880.7)	30 730.3 (24 141.8–38 242.1)	1757.7 (1382.7–2184.6)	0.5 (0.4, 0.6)
Southeast Asia	6303.9 (5013–7638.5)	1904.3 (1511.8–2313)	11 803.2 (9543.4–14 276)	1652.6 (1348.2–1979.1)	−0.5 (−0.7, −0.4)
Southern Latin America	782 (606.6–981.7)	1646.6 (1275.2–2070.5)	1239.4 (957.7–1559.7)	1674.5 (1295.1–2119.2)	0 (0, 0.1)
Southern Sub-Saharan Africa	288.8 (227.3–357.1)	701.4 (552.8–863.4)	546.1 (430.3–679.1)	725.5 (574.2–893.3)	0.1 (0.1, 0.2)
Tropical Latin America	1292.4 (1038.5–1577.5)	1034.7 (833.8–1253.9)	2436.9 (1976.5–2926)	969.9 (789.4–1165.8)	−0.2 (−0.3, −0.2)
Western Europe	6935.4 (5478.8–8576.8)	1490.9 (1183–1846.7)	8805.5 (7036.9–10 873.5)	1490.2 (1181.4–1829.2)	0.1 (0, 0.2)
Western Sub-Saharan Africa	868.8 (691.1–1065.4)	689 (543.9–847.5)	2176.6 (1727.9–2667.4)	735.8 (579.3–902.5)	0.3 (0.2, 0.3)

AAPC = average annual percentage change; ASIR = age-standardized incidence rate; CI = confidence interval; GBD = Global Burden of Disease Study 2019; SDI = Socio-Demographic Index.

When analyzed by SDI quintile, high-middle SDI countries demonstrated the highest ASIR of urolithiasis in 2019 (1576.4; 95% CI [1268.9–1918.4]), while low SDI countries demonstrated the lowest ASIR (981.9; 95% [771.3–1212.3]; Table 1). Of the 21 GBD regions, Eastern Europe demonstrated the highest ASIR of urolithiasis per 100 000 population (4433.7; 95% CI [3542.5–5414.7]) in 2019, substantially higher than the second highest region South Asia (1757.7; 95% CI [1382.7–2184.6]). Eastern Sub-Saharan Africa (565.7; 95% CI [444.3–692.3]) and Central Sub-Saharan Africa (575.4; 95% CI [446.6–711.2]) demonstrated the two lowest ASIRs in 2019 (Table 1).

### 3.2 Trends in incidence

**Fig. 1:**
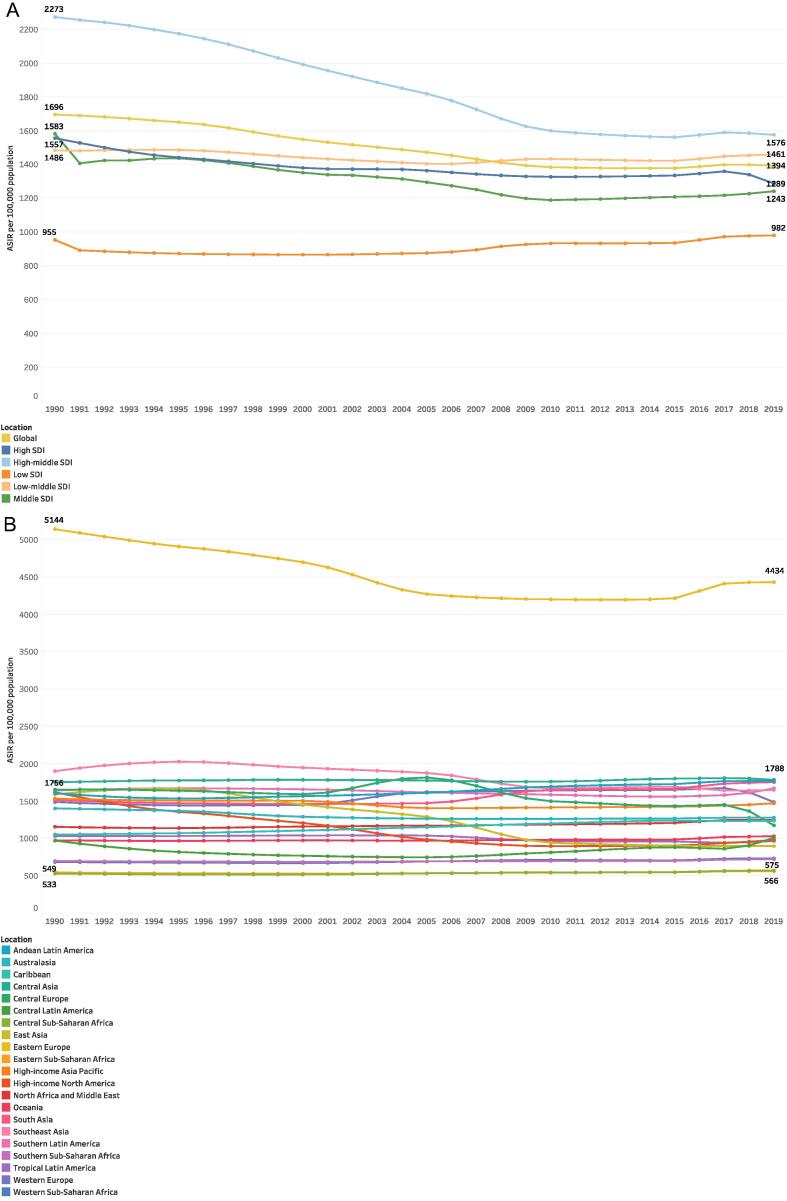
(A) ASIR per 100 000 population globally and by SDI quintile from 1990 to 2019. (B) ASIR per 100 000 population for 21 GBD regions from 1990 to 2019. ASIRs in 1990 and 2019 from Eastern Europe, Central Asia, Eastern Sub-Saharan Africa, and Central Sub-Saharan Africa are shown. ASIR = age-standardized incidence rate; GBD = Global Burden of Disease Study 2019; SDI = Socio-Demographic Index.

Globally, the ASIR of urolithiasis decreased from 1696.2 (95% CI, 1358.1–2078.1) cases per 100 000 population in 1990 to 1394.0 (95% CI, 1126.4–1688.2) cases per 100 000 population in 2019 (Table 1 and Fig. 1). When analyzed by SDI quintile, high-middle SDI countries demonstrated the highest ASIR across all study years with a substantial decrease from 1990 to 2019, while low SDI countries demonstrated the lowest ASIR of urolithiasis, increasing slightly over the study period (Table 1 and Fig. 1).

**Fig. 2:**
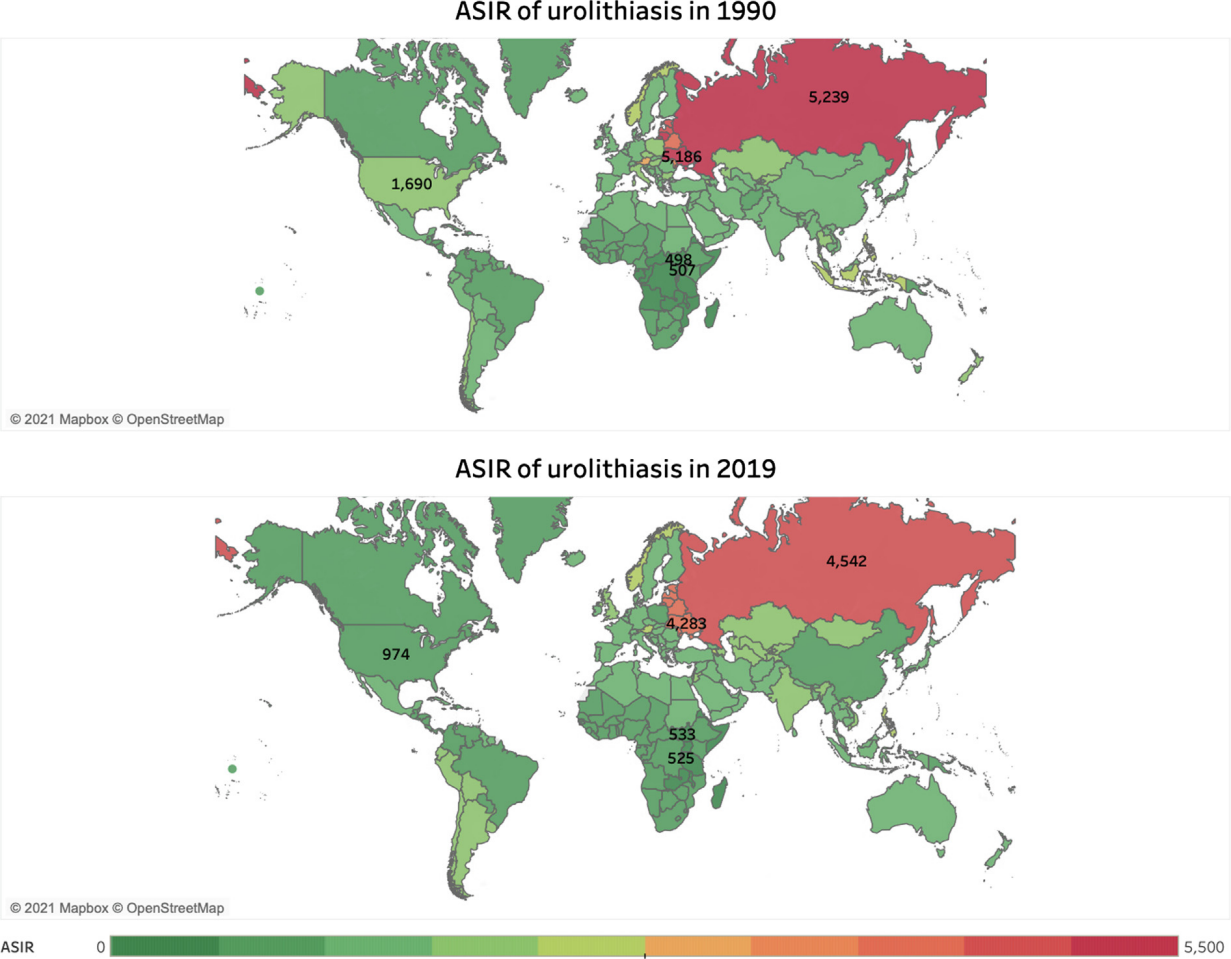
ASIR of urolithiasis in 1990 in 204 countries and that in 2019 in 204 countries and territories. Note that in the published GBD results extracted for this study, Taiwan was incorporated into China and is therefore not separately indicated in these figures. ASIR = age-standardized incidence rate; GBD = Global Burden of Disease Study 2019.

**Fig. 3:**
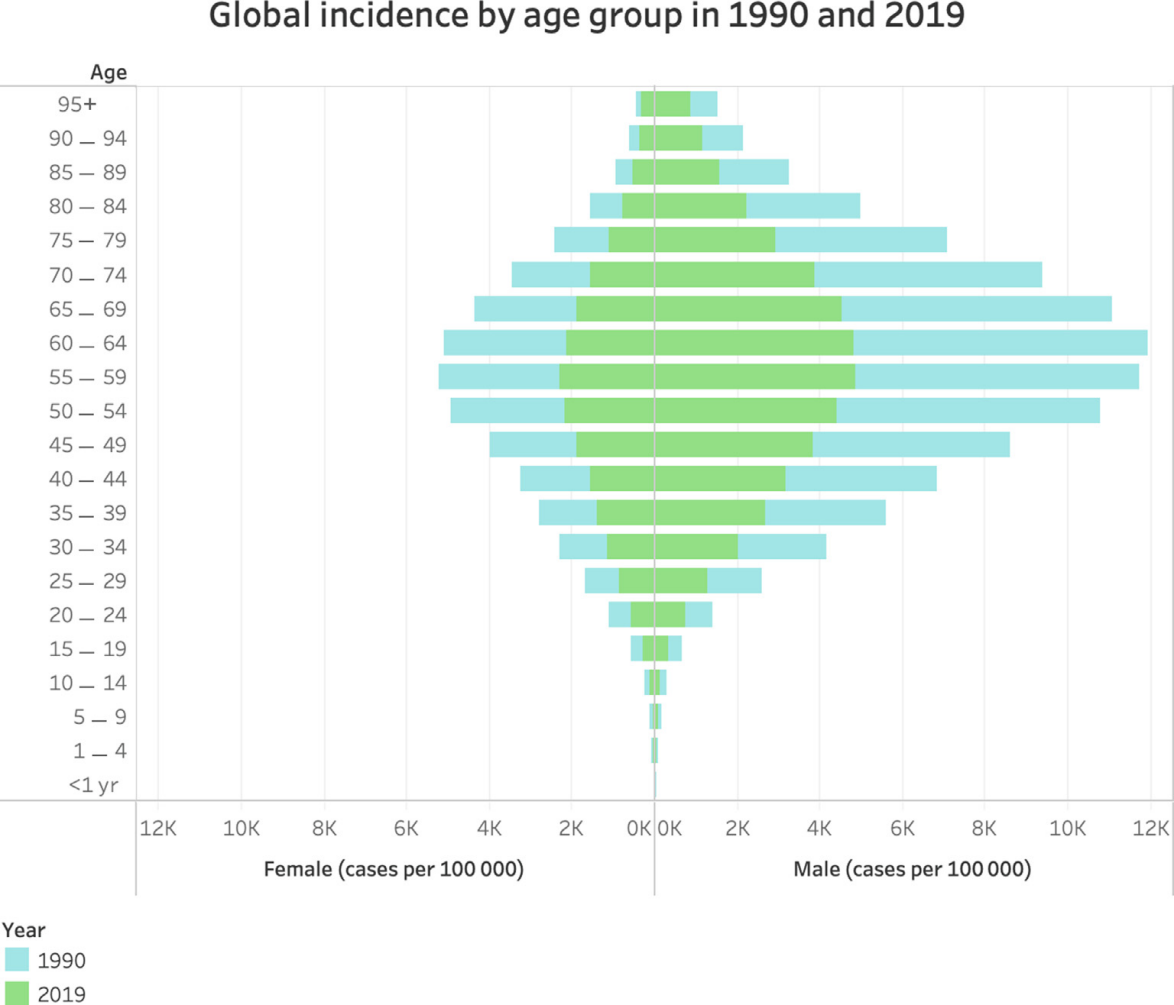
Global incidence rate of urolithiasis by age group and gender for 1990.

When analyzed by GBD region, Eastern Europe demonstrated the highest ASIRs of urolithiasis for all regions from 1990 to 2019, not falling below 4000 cases per 100 000 population throughout the study period (Table 1 and Fig. 1). A country map of ASIRs in 1990 and 2019 is shown in Figure 2. The global age composition of urolithiasis incidence for 1990 and 2019 is shown in Figure 3. Males demonstrated higher rates of global incidence for all age groups in both 1990 and 2019, with the highest rates occurring from ages 50 to 69 yr for both males and females across both years.

### 3.3 Change in incidence

**Fig. 4:**
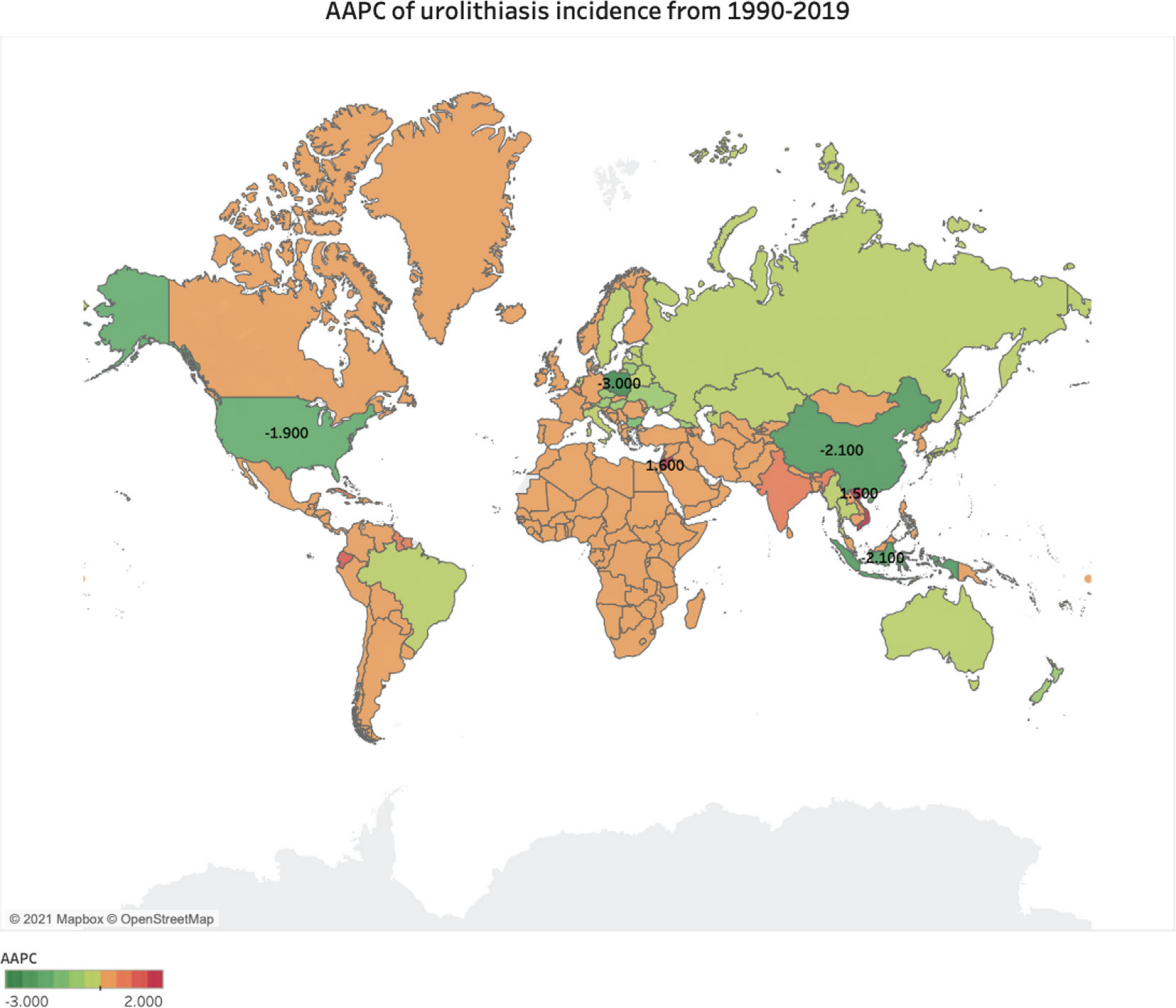
AAPC of urolithiasis incidence from 1990 to 2019 in 204 countries. AAPC = average annual percentage change.

Globally, there was an AAPC of −0.7 (95% CI, −0.8, −0.6; Table 1) from 1990 to 2019, representing a decreasing ASIR. When comparing countries, Poland (−3.0), China (−2.1), Indonesia (−2.1), and the USA (−1.9) demonstrated the most negative AAPC values, while Jordan (1.6) and Vietnam (1.5) demonstrated the highest AAPC values over the study period (Fig. 4).

**Fig. 5:**
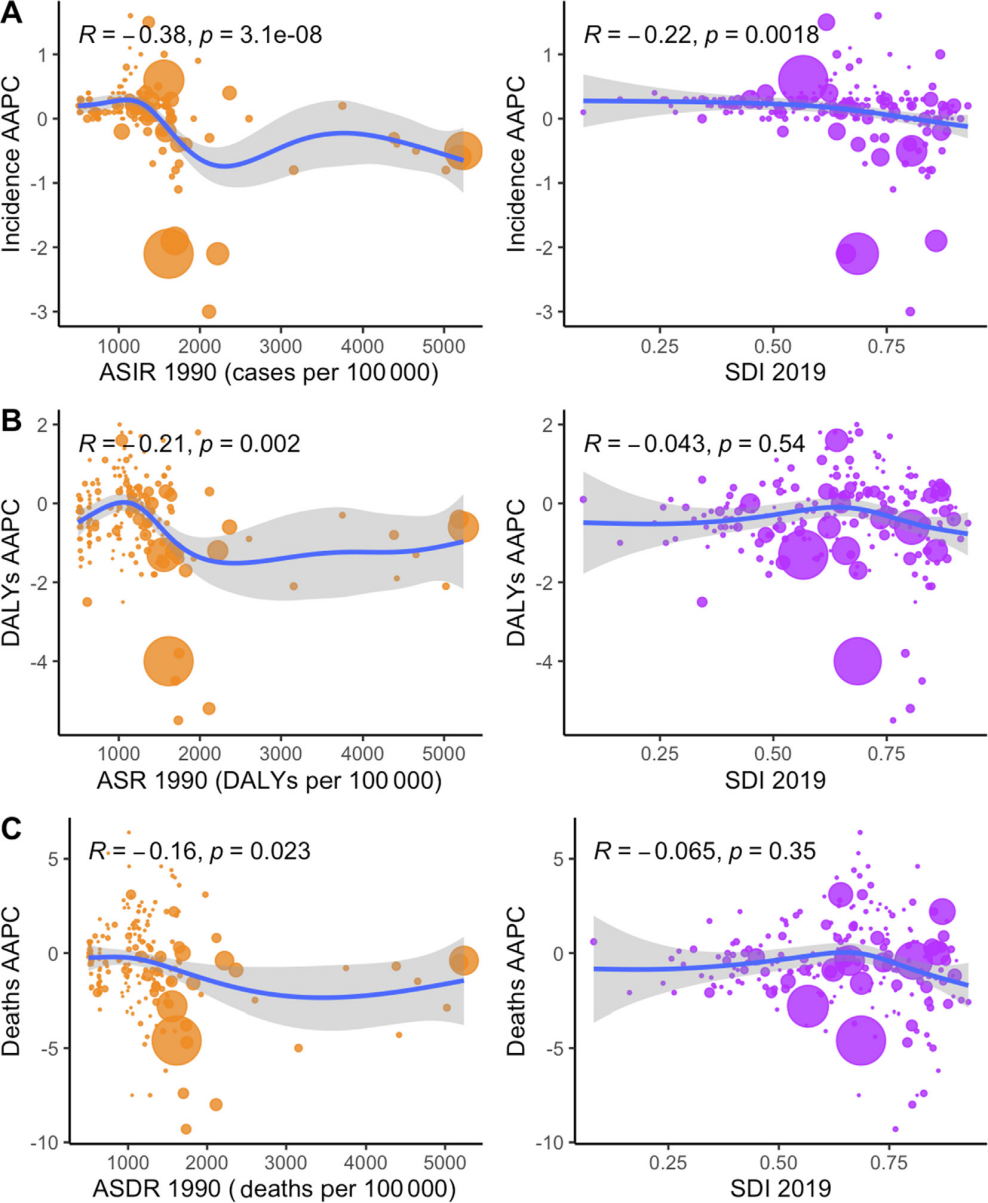
(A) Relationship of AAPC of incidence with ASIR in 1990 and SDI in 2019. (B) Relationship of AAPC of DALYs with ASR of DALYs in 1990 and SDI in 2019. (C) Relationship of AAPC of deaths with ASDR in 1990 and SDI in 2019. Pearson’s *R* correlation coefficient and *p* value of significance are displayed in the top left corner. Size of circle is determined by total cases, DALYs, and deaths in each respective year for A, B, and C, respectively. AAPC = average annual percentage change; ASDR = age-standardized death rate; ASIR = age-standardized incidence rate; ASR = age-standardized rate; DALY = disability-adjusted life year; GBD = Global Burden of Disease Study 2019; SDI = Socio-Demographic Index.

When analyzed by SDI quintile, high-middle SDI (−1.3; 95% CI [−1.3, −1.2]) demonstrated the greatest decrease over the study period, followed by middle SDI (−0.8; 95% CI [−1.1, −0.5]) and high SDI (−0.6; 95% CI [−0.8, −0.5]). Low SDI remained stagnant (0; 95% CI [−0.1, 0]), while low-middle SDI demonstrated the only significant positive increase over the study period (0.1; 95% CI [0, 0.3]; Table 1). Of the 21 GBD regions, East Asia (−2.0; 95% CI [−2.2, −1.8]) demonstrated the greatest decrease over the study period, followed by high-income North America (−1.7; 95% CI [−1.8, −1.6]), and Central Europe (−1.7; 95% CI [−1.8, −1.6]). The Caribbean (0.6; 95% CI [0.5, 0.6]) demonstrated the greatest AAPC over the study period, followed by South Asia (0.5; 95% CI [0.4, 0.6]) and Andean Latin America (0.4; 95% CI [0.3, 0.4]; Table 1). AAPC was significantly negatively correlated with ASIR in 1990 (*R* = −0.38, *p* = 0.000), as well as SDI in 2019 (*R* = −0.22, *p* = 0.002; Fig. 5A).

### 3.4 Change in DALYs

Over the study period, total DALYs attributed to urolithiasis increased globally (Supplementary Table 1). The ASR per 100 000 population of DALYs attributed to urolithiasis, however, decreased from 11.8 (95% CI [8.6–14.4]) in 1990 to 7.4 (95% CI [5.8–9.0]) in 2019 with an AAPC of −1.6 (95% CI [−1.7, −1.4]). Jamaica had the highest AAPC of DALYs for all countries and territories at 2.0 (95% CI [1.4–2.6]) over the study period, while Bulgaria demonstrated the biggest decreasing trend at −5.5 (95% CI [−6.1, −4.9]). AAPCs of DALYs for all countries were weakly negatively correlated with ASIR in 1990 (*R* = −0.21, *p* = 0.002) and were not significantly correlated with SDI in 2019 (*R* = −0.043, *p* = 0.54; Fig. 5B).

When analyzed by SDI quintile, all five quintiles had a negative AAPC, with high-middle SDI demonstrating the most negative AAPC at −2.0 (95% CI [−2.5, −1.6]). For GBD regions, tropical Latin America demonstrated the highest AAPC during the study period at 1.6 (95% CI [1.4, 1.8]). East Asia demonstrated the largest decreasing AAPC over the study period (−3.9; 95% CI [−4.1, −3.7]) followed by Central Europe (−3.7; 95% CI [−3.9,−3.5]). Eastern Europe demonstrated the highest ASR of DALYs throughout the study period, although the AAPC was negative (−0.6; 95% [−1.2, 0]; Supplementary Table 1).

### 3.5 Change in deaths

Similarly, total deaths attributed to urolithiasis increased over the study period (Supplementary Table 2). ASDR attributed to urolithiasis, however, also decreased from 0.3 (95% CI [0.2–0.37]) per 100 000 in 1990 to 0.17 (95% CI [0.14–0.21]) per 100 000 in 2019, with an AAPC of −2.0 (95% CI [−2.2, −1.8]). Jamaica demonstrated the highest AAPC of deaths at 6.4 (95% CI [5.2–7.6]), followed by Costa Rica at 5.3 (95% CI [4.2–6.5]). Bulgaria demonstrated the most negative AAPC at −9.3 (95% CI [−10.1, −8.4]), followed by Poland at −8.0 (95% CI [−8.5, −7.4]). AAPCs of deaths for all countries were weakly correlated with ASDR in 1990 (*R* = −0.16, *p* = 0.023) and not significantly correlated with SDI in 2019 (*R* = −0.065, *p* = 0.35; Fig. 5C).

When analyzed by SDI quintile, all quintiles demonstrated a negative AAPC, with high-middle SDI demonstrating the most negative AAPC at −2.5 (95% CI [−3.2, −1.9]). For GBD regions, tropical Latin America demonstrated the highest AAPC at 3.1 (95% CI [2.6–3.6]), followed by high-income Asia Pacific (1.8; 95% CI [1.3–2.2]) and Central Asia (1.8; 95% CI [1.0–2.7]). Alternatively, Central Europe demonstrated the most negative AAPC at −6.2 (95% CI [−6.8,−5.8]), followed by East Asia at −4.5 (95% CI [−4.8,−4.1]). Despite this, East Asia demonstrated the highest ASDR of all regions in both 1990 and 2019 (Supplementary Table 2).

## 4 Discussion

This study presents a comprehensive picture of the trends and patterns in incidence, DALYs, and deaths attributed to urolithiasis worldwide. We found that the gross global burden in terms of total cases, deaths, and mortality has increased since 1990, consistent with previous literature [21], while ASRs have decreased globally. These trends, however, are not consistent across countries, sociodemographic categories, and geographic regions.

The burden of disease is shared unequally. Three countries, India, China, and the Russian Federation, were burdened by nearly half of global incident cases in 2019. India itself had the burden of over one-fifth of global incident cases. These three countries also face the greatest number of urolithiasis-attributable DALYs and deaths. As population size is a likely factor in the gross burden faced by these countries, some policymakers may prefer to understand these results in terms of rates. Globally, there were 1394 incident cases per 100 000 population in 2019, a decrease from 1696.2 per 100 000 in 1990. The Russian Federation demonstrates the highest ASIR (4541.9 per 100 000) and the second-highest ASR of DALYs (24.7), second to Armenia (33.3). Armenia also had the greatest rates of deaths due to urolithiasis.

The findings of this study are consistent with the epidemiologic transitional model [22]. When examining incidence by SDI quintile, high-middle SDI countries demonstrated the highest ASIR, while low SDI countries had the lowest ASIR in 2019. Incidence rates in low-middle and low SDI quintiles have remained stagnant or have even increased, while those in the middle, high-middle, and high SDI quintiles have seen significant decreases over the study period. Similarly, this study demonstrated regional variation in incidence, with countries in East Asia, high-income North America, and Central Europe demonstrating significant decreases, while regions such as the Caribbean, South Asia, and Andean Latin America demonstrated significant increases.

Globally, incidence is higher in males than in females, and incidence is highest in the 50–69-yr age range. While there is a negative global AAPC of −0.7, several countries and regions also demonstrated positive AAPCs. The global ASR of DALYs likewise decreased with an AAPC of −1.6, and all SDI regions had a negative AAPC. Similarly, the ASDR decreased with an AAPC of −2.0 globally between 1990 and 2019, with all SDI regions demonstrating a negative AAPC. However, total DALYs and deaths attributable to urolithiasis have increased globally, and there is variation by GBD study region, with some regions demonstrating positive AAPCs for DALYs and/or deaths.

Previous studies have suggested that the probability of stone formation varies geographically and socioeconomically across the world [9], [23]. The trends in incidence and burden by location and national SDI status, as shown in this study, can help elucidate the relationship between stone formation and climate, diet, country development level, and other factors suggested to play a role [4], [5], [8], [9], [24], [25], [26], [27]. A thorough understanding of the cause and effect relationship between these factors and urolithiasis merits further study.

It is important to note that urolithiasis carries substantial risks of its own. Those who develop urolithiasis are at 1.3 times greater risk of developing diabetes mellitus, 1.5 times greater risk of developing hypertension, as well as two times greater risk of developing metabolic syndrome and thus two to four times as likely to develop cardiovascular disease [28], [29], [30]. These risks further necessitate preventive policy.

Limitations of this study first and foremost include the limitations of the GBD study itself [11]. Primary data were collected from censuses, household survey, civil registration and vital statistics, satellite imaging, and several other sources. Hence, one of several limitations of the study is varying quality of obtained data and, where primary data were not available, the predictive value of modeling efforts. Additionally, urolithiasis types were not distinguished by the GBD study and may differ by regions based on the variables previously mentioned. Therefore, further research into epidemiologic trends of urolithiasis in different parts of the genitourinary tract and of different chemical compositions requires investigation. Additionally, a linear strategy was used to model ASIRs in different regions and SDI quintiles, when, in actuality, some groups may demonstrate an underlying nonlinear relationship. Finally, risk factors related to diet or climate may be related to trends found in this analysis, but more specific proxy variables were unavailable.

## 5 Conclusions

In conclusion, total cases, DALYs, and deaths attributed to urolithiasis have increased globally since 1990, while the ASRs of these measures have decreased. Importantly, ASIR in low SDI countries is increasing. With the substantial burden of disease attributed to urolithiasis, global and national strategies to address urolithiasis prevention and treatment are necessary. The distribution and trends regarding incidence, deaths, and DALYs analyzed and presented in this study can help inform policy to better address country-specific needs moving forward.

  ***Author contributions*:** Jacob Lang had full access to all the data in the study and takes responsibility for the integrity of the data and the accuracy of the data analysis.

*Study concept and design*: Lang, Narendrula, Ekwenna.

*Acquisition of data*: Lang, Narendrula.

*Analysis and interpretation of data*: Lang, Narendrula.

*Drafting of the manuscript*: Lang, Narendrula.

*Critical revision of the manuscript for important intellectual content*: All authors.

*Statistical analysis*: Lang, Narendrula.

*Obtaining funding*: None.

*Administrative, technical, or material support*: None.

*Supervision*: Ekwenna.

*Other*: None.

  ***Financial disclosures:*** Jacob Lang certifies that all conflicts of interest, including specific financial interests and relationships and affiliations relevant to the subject matter or materials discussed in the manuscript (eg, employment/affiliation, grants or funding, consultancies, honoraria, stock ownership or options, expert testimony, royalties, or patents filed, received, or pending), are the following: None.

  ***Funding/Support and role of the sponsor*:** None.
