# Supplementary material for: Global Trends in Incidence and Burden of Urolithiasis from 1990 to 2019: An Analysis of Global Burden of Disease Study Data
Source: Eur Urol Open Sci. 2022 Jan 3;35:37–46. doi: 10.1016/j.euros.2021.10.008 (PMC8738898; doi:10.1016/j.euros.2021.10.008)
Supplement: Supplementary data 1 [file mmc1.docx]

Supplementary Tables

| **Group** | **1990** | | **2019** | | **1990-2019** |  |
| --- | --- | --- | --- | --- | --- | --- |
|  | **Total DALYs,  Number x 10^3, (95% CI)** | **ASR of DALYs per 100,000 (95% CI)** | **Total DALYs,  Number x 10^3, (95% CI)** | **ASR of DALYs per 100,000 (95% CI)** | **AAPC (95% CI)** |  |
|  |  |  |  |  |  |  |
| **Global** | 516.73 (374.13-635.72) | 11.75 (8.57-14.39) | 604.31 (477.35-745.19) | 7.35 (5.82-9.04) | -1.6 (-1.7,-1.4) |  |
| **SDI** |  |  |  |  |  |  |
| High SDI | 64.65 (49.63-81.81) | 6.68 (5.07-8.53) | 80.26 (62.62-100.6) | 5.3 (3.99-6.75) | -0.8 (-0.9,-0.7) |  |
| High-middle SDI | 164 (133.14-197.07) | 14.68 (11.99-17.61) | 152.52 (121.89-187.14) | 7.94 (6.33-9.79) | -2 (-2.5,-1.6) |  |
| Middle SDI | 158.77 (101.73-198.11) | 12.91 (8.23-16.13) | 194.31 (150.5-240.02) | 7.49 (5.81-9.17) | -1.9 (-2,-1.8) |  |
| Low-middle SDI | 104.78 (56.84-135.55) | 13.86 (7.44-18.79) | 135.96 (97.54-173.9) | 8.85 (6.24-11.25) | -1.5 (-1.7,-1.3) |  |
| Low SDI | 24.35 (16.93-33.23) | 7.6 (5.21-10.43) | 40.9 (29.58-54.79) | 5.88 (4.33-7.87) | -0.9 (-1,-0.7) |  |
| **Region** |  |  |  |  |  |  |
| Andean Latin America | 1.72 (1.19-2.32) | 6.32 (4.46-8.49) | 3.93 (2.83-5.24) | 6.4 (4.62-8.44) | 0.1 (-0.1,0.2) |  |
| Australasia | 1.78 (1.43-2.19) | 7.78 (6.25-9.65) | 2.18 (1.67-2.8) | 5.37 (4.04-6.95) | -1.2 (-1.4,-1.1) |  |
| Caribbean | 1.93 (1.48-2.48) | 6.61 (5.13-8.3) | 4.16 (3.21-5.32) | 8.14 (6.26-10.45) | 0.8 (0.4,1.2) |  |
| Central Asia | 5.72 (4.23-7.64) | 10.83 (8.05-14.57) | 9.6 (7.57-12) | 12.42 (9.88-15.89) | 0.6 (0,1.2) |  |
| Central Europe | 19.39 (15.94-26.32) | 13.51 (11.06-18.26) | 7.43 (5.68-9.61) | 4.52 (3.4-5.95) | -3.7 (-3.9,-3.5) |  |
| Central Latin America | 8.83 (7.4-10.52) | 8.21 (6.86-9.78) | 20.59 (16.74-26.31) | 8.24 (6.7-10.57) | 0.1 (0,0.3) |  |
| Central Sub-Saharan Africa | 1.63 (1.04-2.63) | 5.02 (3.11-7.83) | 3.29 (2.19-4.73) | 4.33 (2.77-6.34) | -0.5 (-0.6,-0.5) |  |
| East Asia | 162.13 (96.12-200.23) | 16.61 (9.81-20.36) | 107.56 (84.19-134.39) | 5.35 (4.17-6.69) | -3.9 (-4.1,-3.7) |  |
| Eastern Europe | 80.93 (66.13-97.91) | 29.44 (24.01-35.53) | 72.97 (58.62-89.34) | 23.61 (18.69-29.23) | -0.6 (-1.2,0) |  |
| Eastern Sub-Saharan Africa | 6.74 (4.43-11.11) | 6.37 (4.24-10.05) | 10.68 (6.94-16.21) | 4.83 (3.18-7.5) | -0.9 (-1,-0.9) |  |
| High-income Asia Pacific | 10.96 (7.71-14.82) | 5.48 (3.91-7.35) | 18.89 (14.52-23.73) | 5.7 (4.18-7.45) | 0.2 (0,0.3) |  |
| High-income North America | 19.75 (14.71-25.63) | 6.16 (4.55-8.08) | 23.41 (18.77-29.01) | 4.52 (3.59-5.63) | -1 (-1.2,-0.9) |  |
| North Africa and Middle East | 9.83 (6.71-13.52) | 3.95 (2.73-5.46) | 23.56 (15.9-32.82) | 4.1 (2.82-5.65) | 0.1 (0.1,0.2) |  |
| Oceania | 0.3 (0.17-0.43) | 7.31 (3.99-10.28) | 0.62 (0.38-0.87) | 6.25 (3.86-8.8) | -0.5 (-0.6,-0.5) |  |
| South Asia | 84.28 (53.63-113.44) | 11.47 (7.08-16.51) | 135.5 (97.92-180.67) | 8.33 (6-11.1) | -1.1 (-1.5,-0.8) |  |
| Southeast Asia | 53.03 (24.4-71.29) | 17.17 (7.93-23.59) | 85.75 (46.07-109.02) | 13.06 (6.92-16.54) | -0.9 (-1.2,-0.7) |  |
| Southern Latin America | 2.48 (1.67-3.45) | 5.23 (3.53-7.3) | 4.15 (2.84-5.76) | 5.54 (3.78-7.74) | 0.2 (0,0.3) |  |
| Southern Sub-Saharan Africa | 1.52 (1.11-1.92) | 3.71 (2.85-4.68) | 2.5 (1.87-3.26) | 3.46 (2.63-4.44) | -0.2 (-0.5,0) |  |
| Tropical Latin America | 6.46 (5.11-8.09) | 5.43 (4.34-6.76) | 21.19 (16.92-29.89) | 8.55 (6.84-12.13) | 1.6 (1.4,1.8) |  |
| Western Europe | 32.62 (25.4-41.16) | 6.55 (5.04-8.41) | 36.84 (27.51-47.44) | 5.55 (3.99-7.28) | -0.5 (-0.6,-0.4) |  |
| Western Sub-Saharan Africa | 4.71 (2.98-6.51) | 4.12 (2.55-5.79) | 9.52 (6.55-12.65) | 3.61 (2.42-4.79) | -0.4 (-0.5,-0.4) |  |

**Supplementary Table 1:** Total DALYs, ASRs of DALYs, and AAPC of DALYs attributed to Urolithiasis in 1990 and 2019 globally as well as among SDI quintiles and 21 GBD regions.

| **Group** | **1990** | | **2019** | | **1990-2019** |  |
| --- | --- | --- | --- | --- | --- | --- |
|  | **Total Deaths,  Number x 10^3, (95% CI)** | **ASDR per 100,000 (95% CI)** | **Total Deaths,  Number x 10^3, (95% CI)** | **ASDR per 100,000 (95% CI)** | **AAPC (95% CI)** |  |
|  |  |  |  |  |  |  |
| **Global** | 11.34 (7.28-13.78) | 0.3 (0.2-0.37) | 13.28 (10.62-16.27) | 0.17 (0.14-0.21) | -2 (-2.2,-1.8) |  |
| **SDI** |  |  |  |  |  |  |
| High SDI | 1.33 (1.1-1.63) | 0.13 (0.1-0.16) | 2.24 (1.77-3.03) | 0.1 (0.08-0.14) | -0.7 (-0.9,-0.5) |  |
| High-middle SDI | 3.79 (2.98-4.37) | 0.37 (0.29-0.43) | 3.51 (3.02-4.34) | 0.18 (0.15-0.22) | -2.5 (-3.2,-1.9) |  |
| Middle SDI | 3.46 (1.68-4.43) | 0.37 (0.18-0.48) | 4.11 (2.82-5.27) | 0.18 (0.13-0.24) | -2.4 (-2.5,-2.3) |  |
| Low-middle SDI | 2.29 (0.83-3.32) | 0.41 (0.15-0.62) | 2.75 (1.4-3.66) | 0.22 (0.11-0.29) | -2.1 (-2.4,-1.8) |  |
| Low SDI | 0.46 (0.27-0.69) | 0.22 (0.12-0.36) | 0.66 (0.42-0.96) | 0.15 (0.1-0.23) | -1.2 (-1.3,-1.2) |  |
| **Region** |  |  |  |  |  |  |
| Andean Latin America | 0.01 (0.01-0.02) | 0.07 (0.04-0.1) | 0.04 (0.02-0.05) | 0.06 (0.03-0.09) | -0.3 (-0.6,0.1) |  |
| Australasia | 0.05 (0.04-0.06) | 0.2 (0.16-0.24) | 0.05 (0.04-0.07) | 0.1 (0.08-0.14) | -2.3 (-2.7,-1.9) |  |
| Caribbean | 0.03 (0.03-0.04) | 0.13 (0.1-0.17) | 0.09 (0.07-0.12) | 0.18 (0.13-0.24) | 1.2 (0.3,2.1) |  |
| Central Asia | 0.12 (0.08-0.18) | 0.27 (0.19-0.42) | 0.24 (0.19-0.36) | 0.44 (0.32-0.69) | 1.8 (1,2.7) |  |
| Central Europe | 0.59 (0.51-0.88) | 0.42 (0.36-0.62) | 0.14 (0.11-0.19) | 0.07 (0.05-0.08) | -6.2 (-6.7,-5.8) |  |
| Central Latin America | 0.18 (0.15-0.22) | 0.22 (0.17-0.26) | 0.49 (0.39-0.69) | 0.21 (0.17-0.29) | -0.1 (-0.2,0.1) |  |
| Central Sub-Saharan Africa | 0.03 (0.02-0.05) | 0.14 (0.06-0.23) | 0.06 (0.03-0.1) | 0.11 (0.05-0.19) | -0.8 (-0.8,-0.7) |  |
| East Asia | 4.07 (1.85-5.14) | 0.55 (0.25-0.69) | 2.7 (1.83-3.66) | 0.15 (0.1-0.2) | -4.5 (-4.8,-4.1) |  |
| Eastern Europe | 1.8 (1.54-2.31) | 0.65 (0.56-0.83) | 1.89 (1.55-2.28) | 0.55 (0.45-0.66) | -0.5 (-1.5,0.5) |  |
| Eastern Sub-Saharan Africa | 0.14 (0.08-0.24) | 0.23 (0.13-0.37) | 0.23 (0.13-0.41) | 0.2 (0.12-0.34) | -0.5 (-0.6,-0.3) |  |
| High-income Asia Pacific | 0.12 (0.09-0.15) | 0.07 (0.05-0.09) | 0.7 (0.5-0.96) | 0.11 (0.09-0.15) | 1.8 (1.3,2.2) |  |
| High-income North America | 0.33 (0.27-0.41) | 0.09 (0.07-0.11) | 0.62 (0.51-0.85) | 0.09 (0.08-0.13) | 0.1 (-0.3,0.4) |  |
| North Africa and Middle East | 0.05 (0.03-0.08) | 0.03 (0.01-0.06) | 0.12 (0.07-0.15) | 0.03 (0.02-0.04) | 0.3 (0,0.7) |  |
| Oceania | 0.01 (0-0.01) | 0.17 (0.06-0.28) | 0.01 (0-0.01) | 0.12 (0.05-0.2) | -1.1 (-1.3,-1) |  |
| South Asia | 1.62 (0.74-2.58) | 0.33 (0.15-0.56) | 2.08 (1.22-3.08) | 0.16 (0.1-0.25) | -2.4 (-3.2,-1.6) |  |
| Southeast Asia | 1.21 (0.32-1.78) | 0.5 (0.15-0.74) | 2.18 (0.7-2.95) | 0.4 (0.13-0.55) | -0.7 (-1,-0.4) |  |
| Southern Latin America | 0.01 (0.01-0.02) | 0.03 (0.02-0.04) | 0.03 (0.03-0.04) | 0.04 (0.03-0.05) | 1.5 (0.9,2.1) |  |
| Southern Sub-Saharan Africa | 0.02 (0.01-0.02) | 0.05 (0.04-0.07) | 0.03 (0.02-0.04) | 0.05 (0.04-0.07) | -0.2 (-0.9,0.6) |  |
| Tropical Latin America | 0.09 (0.07-0.11) | 0.1 (0.07-0.12) | 0.55 (0.43-0.93) | 0.23 (0.18-0.39) | 3.1 (2.6,3.6) |  |
| Western Europe | 0.77 (0.57-0.87) | 0.13 (0.1-0.15) | 0.88 (0.73-1.24) | 0.08 (0.07-0.12) | -1.5 (-1.8,-1.3) |  |
| Western Sub-Saharan Africa | 0.08 (0.03-0.13) | 0.11 (0.04-0.19) | 0.12 (0.05-0.18) | 0.08 (0.03-0.11) | -1.2 (-1.3,-1.1) |  |

**Supplementary Table 2:** Total Deaths, ASDRs, and AAPC of Deaths attributed to Urolithiasis in 1990 and 2019 globally as well as among SDI quintiles and 21 GBD regions.

| **Term** | **Definition** |
| --- | --- |
| Age-Standardized Rate (ASR) | An ASR is a weighted average of the age-specific rates, where the weights are a proportional distribution of a standard population age groups. Thus, age is removed as a confounding variable when comparing ASRs across groups (e.g. countries, regions) using the same standard population. |
| Age-Standardized Incidence Rate (ASIR) | The ASR of Incidence. Incidence rate is the number of new cases of disease (e.g. urolithiasis) occurring per unit of population, per unit time. In this case, per 100,000 population, per year. |
| Age-Standardized Death Rate (ASDR) | The ASR of Death. Death rate is the number of deaths due to a disease (e.g. urolithiasis) occurring per unit of population. In this case, per 100,000 population. |
| ASR of Disability Adjusted Life Years (DALYs) | The ASR of DALYs. DALYs are a measure of the gap in health years of life lived (e.g. with urolithiasis) compared to a standard population. DALYs encompass years of life lost due to premature mortality as well as years of life lived with disability or illness. One DALY is equivalent to healthy year of life lost. The DALY rate is measured in DALYs per 100,000 population. |

**Supplementary Table 3: Definitions of ASR, ASIR, ASDR, and ASR of DALYs, adapted from the Global Burden of Disease and Risk Factors Glossary.^1^**

**References**

1. Lopez AD, Mathers CD, Ezzati M, Jamison DT, Murray CJ. Glossary. 2006. https://www.ncbi.nlm.nih.gov/books/NBK11818/. Accessed August 30, 2021.
